# Surgical decision-making for concomitant tricuspid valve repair in minimally invasive mitral valve surgery

**DOI:** 10.1093/ejcts/ezaf201

**Published:** 2025-07-17

**Authors:** Can Gollmann-Tepeköylü, Paolo Berretta, Marc Gerdisch, Giovanni D Cresce, Jörg Kempfert, Antonios Pitsis, Frank Van Praet, Mauro Rinaldi, Manuel Wilbring, Tristan Yan, Davide Pacini, Torsten Doenst, Antonio Fiore, Nguyen Hoang Dinh, Joseph Lamelas, Pierluigi Stefano, Tom C Nguyen, Nikolaos Bonaros, Marco Di Eusanio

**Affiliations:** Department of Cardiac Surgery, Medical University of Innsbruck, Innsbruck, Austria; Cardiac Surgery Unit, Lancisi Cardiovascular Center, Polytechnic University of Marche, Ancona, Italy; Franciscan Health Indianapolis, Indianapolis, IN, USA; Division of Cardiac Surgery, S. Bortolo Hospital, Vicenza, Italy; Department of Cardiothoracic and Vascular Surgery, German Heart Center Berlin, Berlin, Germany; Cardiac Surgery Department, European Interbalkan Medical Center, Thessaloniki, Greece; Cardiac Surgery Department, Hartcentrum OLV Aalst, Aalst, Belgium; Cardiac Surgery Unit, University of Turin, Turin, Italy; Center for Minimally Invasive Cardiac Surgery, University Heart Center Dresden, Dresden, Germany; Department of Cardiothoracic Surgery, The Royal Prince Alfred Hospital, Sydney, Australia; Cardiac Surgery Department, Sant’Orsola Malpighi Hospital, University of Bologna, Bologna, Italy; Department of Cardiothoracic Surgery, Jena University Hospital, Jena, Germany; Henri Mondor Hospital, University of Paris, Paris, France; University of Medicine and Pharmacy, Ho Chi Minh City, Vietnam; Division of Cardiothoracic Surgery, University of Miami, Miami, FL, USA; Cardiac Surgery Unit, Careggi University Hospital, Firenze, Italy; Baptist Health Miami Cardiac & Vascular Institute, Miami, FL, USA; Department of Cardiac Surgery, Medical University of Innsbruck, Innsbruck, Austria; Cardiac Surgery Unit, Lancisi Cardiovascular Center, Polytechnic University of Marche, Ancona, Italy

**Keywords:** Valvular heart disease, Minimally invasive mitral valve surgery, Concomitant tricuspid valve repair

## Abstract

**OBJECTIVES:**

To identify factors influencing the decision to omit tricuspid valve repair in patients who meet guideline criteria for tricuspid valve repair undergoing minimally invasive mitral valve surgery (MIMVS).

**METHODS:**

A retrospective analysis was conducted using the MIMVS International Registry, covering 7513 patients from 17 centres in Europe USA, Asia and Australia. Of these, 1077 had an indication for tricuspid valve repair. Patients were stratified into two groups: those who underwent tricuspid valve repair (*n* = 910) and those who did not (*n* = 167). Multivariate logistic regression analysis was conducted to identify the factors associated with the decision to perform tricuspid valve repair.

**RESULTS:**

Patients who received tricuspid valve repair were older (72 vs 67 years, *P* < 0.001), more often female (53.8% vs 39.8%, *P* < 0.001) and had higher rates of atrial fibrillation (70.1% vs 54%, *P* < 0.001). Tricuspid valve repair was associated with longer ICU (48 vs 23 hours, *P* < 0.001) and hospital stays (11 vs 8 days, *P* < 0.001), but 30-day mortality was similar between groups (4.3% for tricuspid valve repair vs 1.8% for no tricuspid valve repair, *P* = 0.2). Patients undergoing tricuspid valve repair had higher EuroSCORE II (2.9 vs 1.6, *P* < 0.001). Key factors for omitting tricuspid valve repair included absence of severe tricuspid regurgitation (odds ratio [OR] 3.31 for moderate tricuspid regurgitation; OR 4.06 for mild tricuspid regurgitation), lower NYHA class (OR 0.61 for NYHA III-IV), and mitral valve disease type (OR 0.38) and institutional practices (SD 0.28).

**CONCLUSIONS:**

Prophylactic indications for concomitant tricuspid valve repair in MIMVS are generally followed. Clinical and institutional factors strongly influence the decision to omit the tricuspid procedure despite guideline recommendations. Adhering to guidelines may improve outcomes by standardizing treatment choices.

## Graphical Abstract

**Figure:**
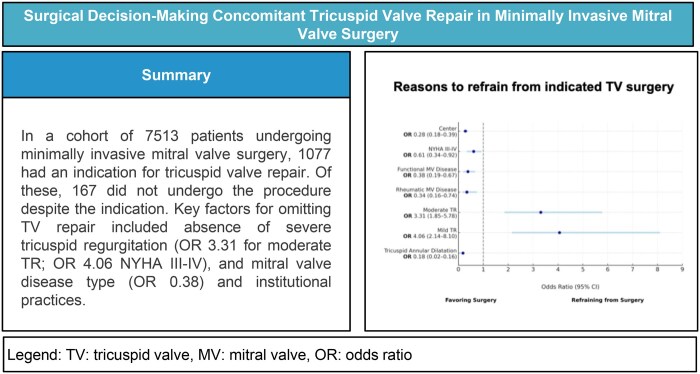

## INTRODUCTION

Minimally invasive mitral valve surgery (MIMVS) has emerged as a preferred approach over traditional open surgery due to its association with reduced invasiveness, shorter hospital stays and faster recovery times [1–3]. The increasing use of MIMVS has prompted integration of concomitant tricuspid valve (TV) repair in patients with significant tricuspid regurgitation (TR). For patients with coexisting TR, concomitant TV repair is often indicated to prevent the progression of TR and its associated adverse outcomes, which include right ventricular dysfunction, liver congestion and decreased long-term survival [4].

According to current guidelines, concomitant TV repair is recommended for severe TR as a class I indication in patients undergoing left-sided valve procedure. In addition and according to the guidelines, concomitant TV repair should be considered in mild or moderate TR with annular dilatation exceeding 40 mm, with the goal of preventing TR progression and potential right-sided heart failure, both of which can worsen postoperative outcomes [5, 6]. Concomitant tricuspid repair might decrease reoperation, disease progression or death, however, at the price of higher permanent pacemaker implantations [7].

Despite these clear indications, several surgeons decide against concomitant TV repair. This decision is often driven by concerns about prolonged operative time and invasiveness, added technical complexity, increased risk for permanent pacemaker implantations and impaired outcomes, particularly in older or high-risk patient populations [8]. For these patients, even minimally invasive approaches carry risks, and the potential benefits of TV repair must be carefully weighed against the likelihood of adverse events associated with extended operative procedures. Institutional practices and individual surgeon preferences further contribute to the variability in adherence to guideline-based indications for TV repair. To which extend the complexity of MIMVS contributes to the decision to omit concomitant tricuspid surgery remains to be determined.

This study aimed to analyse the factors influencing the decision to refrain from concomitant TV repair in patients undergoing MIMVS who had a class I or class IIa indication for TV intervention, as defined by current ESC/EACTS guidelines. While all patients in our cohort received mitral valve (MV) surgery, the key clinical dilemma lies in the omission of guideline-recommended TV repair—particularly in the setting of a minimally invasive approach, where concerns about procedural complexity may impact surgical judgement. Using data from the Mini Mitral International Registry (MMIR), a large, multicentre database, we evaluated patient characteristics, institutional practice patterns and outcomes associated with this decision-making process. By identifying the determinants that lead surgeons to deviate from guideline-directed therapy, we hope to inform clinical strategies that promote better adherence to recommendations and ultimately improve long-term outcomes for patients undergoing MV surgery.

## MATERIALS AND METHODS

### Ethical statement

The study protocol was approved by the local institutional review board of all centres based on the approval of the coordinating centre (n. 2020189, 30 July 2020), and consent of patients was waived.

### Study design and population

**Figure 1:**
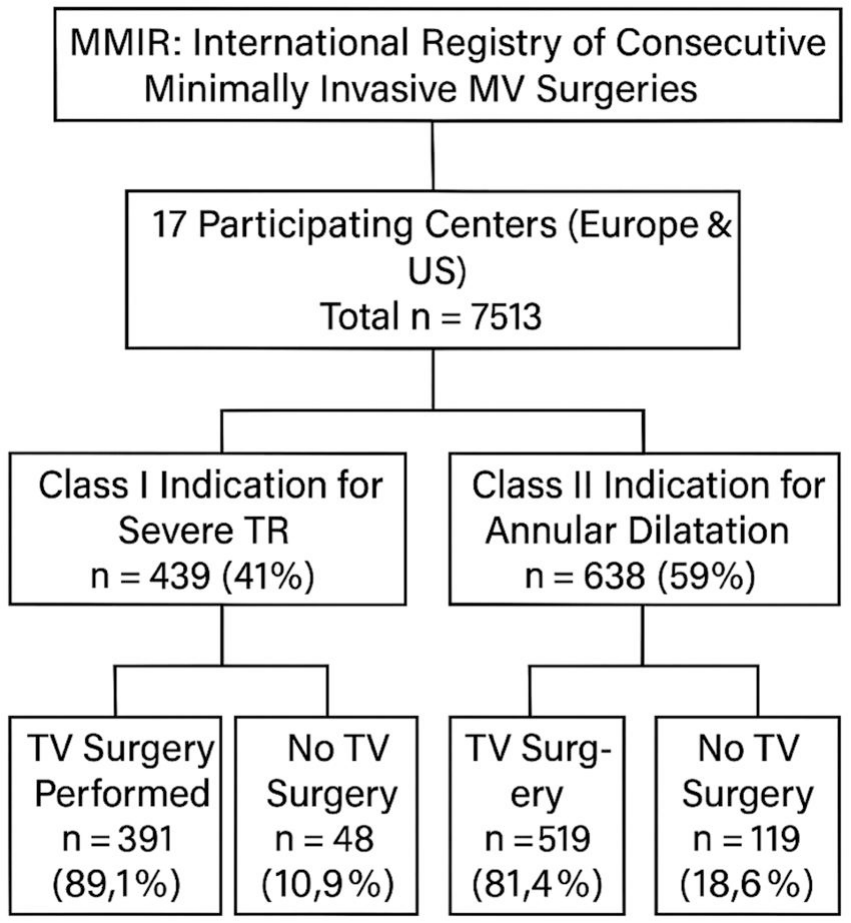
CONSORT diagram. CONSORT diagram illustrating the patient selection process for the study. The diagram details inclusion and exclusion criteria, starting from the total cohort in the MMIR registry to the final study groups with and without concomitant tricuspid valve repair.

In this retrospective, observational cohort study, data from the MMIR were analysed. The MMIR is a multicentre international registry that collects consecutive cases of MIMVS across 17 high-volume centres in Europe, USA, Asia and Australia. The rationale and methods of MMIR were previously reported [9]. At the time of this study, the registry included data from 7513 patients undergoing MIMVS. Among this cohort, 1077 patients were identified as having TV disease with a guideline-based indication for concomitant TV repair. Patients with active endocarditis and concomitant aortic valve and aortic root replacement were excluded. The study cohort was categorized based on indication of TV surgery: 439 patients (41%) had a class I indication for TV repair due to severe TR, while 638 patients (59%) had a class II indication related to tricuspid annular dilatation [5, 6]. Patients were then grouped according to whether they received concomitant TV repair. Of the 1077 patients with an indication for TV intervention, 910 (84.5%) underwent TV repair, whereas 167 (15.5%) did not receive this additional procedure (Fig. 1). This categorization allowed for the assessment of demographic, clinical and institutional factors influencing the decision-making process for performing concomitant TV repair in patients undergoing minimally invasive MV surgery. Based on the assumption that MIMVS is usually performed by experienced mitral and TV surgeons, we hypothesized that decision-making for or against concomitant tricuspid surgery was based on the current institutional policy. Therefore, the factor participating centre as an institution rather than the surgeon was included into the analysis.

### Operative approach

All the procedures were performed through a non-sternotomy minimally invasive approach. The choice of surgical approach was based on the discretion of the surgeon at each participating centre. Patients underwent either direct-vision, video-assisted surgery or totally endoscopic surgery. The choice of approach (direct vision, video-assisted, totally endoscopic or robotic) and cannulation sites (femoral, axillary or ascending aorta) were recorded for each case. Patients undergoing concomitant atrial fibrillation (AFib) surgery, whether performed via ablation or the Maze procedure, were also noted.

### Outcomes and variables

The primary outcome was the decision to perform concomitant TV surgery. Secondary outcomes included in-hospital mortality, incidence of stroke, postoperative delirium, new-onset AFib, need for a permanent pacemaker, ICU and hospital stay, and other perioperative complications such as bleeding and vascular complications. Technical success was defined as successful completion of intended MV treatment (repair or replacement) resulting in a competent valve at the end of the procedure, without mortality, the need for conversion to sternotomy or secondary MV replacement after failed repair. If a patient underwent a priori mitral replacement due to an irreparable valve, this was considered to fulfil the criteria of technical success, unless there were any signs of valve dysfunction such as paravalvular leakage or valve obstruction. The following preoperative characteristics were included: age, sex, NYHA functional class, presence of AFib, MV disease aetiology (degenerative, functional or rheumatic) and EuroSCORE II. Intraoperative variables included cardiopulmonary bypass (CPB) and cross-clamp times, use of cardioplegia or beating heart strategies and technical success rates. Postoperative outcomes included ICU and hospital stay durations and major complications.

### Statistical analysis

Continuous variables were expressed as medians with interquartile ranges (IQRs) and compared between groups using the Mann–Whitney *U*-test. Categorical variables were reported as frequencies and percentages and compared using chi-square or Fisher’s exact tests, as appropriate. Multivariate logistic regression was used to identify factors influencing the decision to refrain from concomitant TV repair. The model was adjusted for potential confounders selected a priori on the basis of their clinical significance that may directly influence the outcome (sex, age, pacemaker (PM), critical preoperative state, redo, urgent/emergent status, renal impairment, NYHA III-IV, left ventiruclar ejection fraction (LVEF) < 50%, pulmonary hypertension, MV disease aetiology, type of functional TR [atrial versus ventricular], grading of TR, TR annular dilatation, surgical approach, centre). Backward stepwise method was used to build the final model. The backward stepwise method was used to build the final model, with a *P* value threshold of 0.10 for exclusion. To assess potential overfitting, an internal validation was performed using a bootstrap approach with 1000 resamples. The results indicated no substantial overfitting, with a shrinkage factor of 1.03. Adjusted odds ratios (ORs) and 95% confidence intervals (CIs) were reported for each predictor. To account for variability across sites, centres were included as random intercept terms in the model. The standard deviation and CIs of the random effect for centre were reported. Multicollinearity was assessed by the variance inflation factor. All analyses were conducted using Statistical Package for Social Sciences version 29.0 (IBM, Chicago, IL) with a significance threshold of *P* < 0.05.

### Data availability

The data underlying this article will be shared on reasonable request to the corresponding author.

## RESULTS

Totally, 1077 patients in our cohort were identified who had an indication of concomitant TV repair during MIVMS. Among these patients, 910 (84.5%) underwent concomitant TV repair during MIMVS, while 167 (15.5%) did not (Table 1). No patients underwent TV replacement. Among patients who received concomitant TV repair (*n* = 910), 391 (43%) had a class I indication (severe TR), and 519 (57%) had a class IIa indication (annular dilatation without severe TR). In contrast, among those who did not undergo TV repair (*n* = 167), only 48 patients (28.7%) had a class I indication, while 119 patients (71.3%) had a class IIa indication. Patients who received TV repair were older, with a median age of 72 years compared to 67 years in those who did not (*P* < 0.001). Female patients were more frequently represented in the TV repair group (53.8% vs 39.8%, *P* < 0.001). Additionally, these patients were more symptomatic, with 68.6% presenting in NYHA functional class III-IV compared to 54.8% in the group without TV repair (*P* = 0.001), suggesting more advanced heart failure in those undergoing concomitant repair. Atrial fibrillation was notably more prevalent in the TV repair group (70.1% vs 54%, *P* < 0.001), and a higher proportion of these patients exhibited degenerative MV disease as the primary aetiology (50.1% vs 11.8%, *P* < 0.001). Patients without TV repair had more frequently ventricular functional TR (46% vs 29.9%, *P* < 0.001), indicating a difference in the nature of tricuspid pathology between groups. Tricuspid annular dilatation was observed more commonly in patients who underwent TV repair (94.3% vs 88.2%, *P* = 0.01), and severe TR was also more prevalent in this group (43% vs 28.7%, *P* = 0.002). Surprisingly, these baseline characteristics highlighted a tendency towards more advanced disease and a higher risk profile in patients selected for concomitant TV repair.

**Table 1:** Baseline characteristics of patients undergoing MIMVS with or without concomitant tricuspid valve repair

	Concomitant tricuspid surgery (*n* = 910)	No tricuspid surgery (*n* = 167)	*P* value
Female	489 (53.8)	66 (39.8)	<0.001
Age, median (IQR)	72 (65–77)	67 (58-76)	<0.001
NYHA III-IV	608 (68.6)	86 (54.8)	0.001
Diabetes	111 (12.2)	20 (12)	1
Smoking	85 (10.2)	24 (14.7)	0.1
Obesity	170 (18.7)	19 (11.7)	0.03
Atrial fibrillation	583 (70.1)	88 (54)	<0.001
Pacemaker	67 (7.4)	7 (4.2)	0.2
Dialysis	12 (1.3)	3 (1.8)	0.7
Chronic lung disease	113 (12.4)	18 (10.9)	0.7
Cerebrovascular arteriopathy	19 (2.1)	3 (2)	1
Peripheral arteriopathy	39 (4.3)	4 (2.4)	0.4
Pulmonary hypertension	555 (61.7)	96 (58.2)	0.4
Previous cardiac surgery	106 (11.6)	15 (9.4)	0.5
Mitral valve disease aetiology			<0.001
Degenerative	429 (50.1)	114 (79.2)	
Functional	293 (34.2)	17 (11.8)	
Rheumatic	91 (10.6)	10 (6.9)	
Other	43 (5)	3 (1.8)	
Tricuspid annular dilatation (≥40mm or >21 mm/m^2^)	725 (94.3)	120 (88.2)	0.01
LVEF ≤ 50%	264 (29.4)	30 (18)	0.002
ES II, median (IQR)	2.9 (1.6–5.5)	1.6 (0.8–3.1)	<0.001

Values represent counts (percentages) or medians (IQR). Statistical significance is denoted for key inter-group comparisons. ES II: EuroSCORE II; IQR: interquartile range; LVEF: Left ventricular ejection fraction.

Operative characteristics demonstrated differences in surgical approach and complexity between the two groups (Table 2). Patients undergoing concomitant TV repair were more likely to have MV replacement rather than repair (30% vs 22%, *P* = 0.03). A higher percentage of these patients underwent concomitant AFib surgery (32.2% vs 16.8%, *P* < 0.001). Median CPB times were slightly longer in the TV repair group (157 vs 146 min, *P* = 0.03), while cross-clamp times were comparable between groups (median 88 min in both groups, *P* = 0.2). Technical success of MV repair was high across both groups, with 97.1% success in the TV repair group and 96% in the no-TV repair group (*P* = 0.4), suggesting that concomitant repair did not compromise effectiveness of MV procedure.

**Table 2:** Operative characteristics among patients with and without tricuspid valve repair, comparing type of mitral valve surgery, concomitant atrial fibrillation surgery, CPB times and technical success rates

	Concomitant tricuspid surgery (*n* = 910)	No tricuspid surgery (*n* = 167)	*P* value
Conversion to full sternotomy	20 (2.2)	5 (3)	0.6
Type of surgery			0.03
Mitral valve repair	631 (69.3)	127 (76)	
Mitral valve replacement	274 (30.1)	37 (22.2)	
Replacement due to unsuccessful repair	5 (0.5)	3 (1.8)	
Concomitant atrial fibrillation surgery	293 (32.2)	28 (16.8)	<0.001
Surgical approach			
Direct vision	396 (43.5)	50 (29.9)	
Video assisted	273 (30.0)	80 (47.9)	
Totally endoscopic	238 (26.2)	36 (21.6)	
Robotic	1 (0.1)	1 (0.6)	
Unknown	2(0.2)		
Repeated x-clamping	13 (1.5)	5 (3.4)	0.2
CPB time (min), median (IQR)	157 (130–195)	146 (118–193)	0.03
X-clamp time (min), median (IQR)	88 (61–119)	88 (69.8–118.2)	0.2
Technical success	846 (97.1)	144 (96)	0.4

CPB: cardiopulmonary bypass; IQR: interquartile range.

Postoperatively, ICU stay was prolonged in the TV repair group, with a median duration of 48 hours compared to 23 hours for those without TV repair (*P* < 0.001) (Table 3). Hospital stays were longer in the concomitant TV group, with a median of 11 days versus 8 days in the group without TV repair (*P* < 0.001). Patients undergoing TV repair experienced higher rates of postoperative delirium (12.1% vs 6.3%, *P* = 0.04) and new-onset AFib (8.2% vs 14.1%, *P* = 0.03). Despite these differences, major complications, including in-hospital mortality, stroke and bleeding, were comparable between groups, with in-hospital mortality rates of 4.3% in the TV repair group and 1.8% in the group without concomitant TV repair (*P* = 0.2).

**Table 3:** In-hospital outcomes of patients undergoing MIMVS with and without tricuspid valve repair, detailing mortality, major complications, ICU and hospital stay durations and specific adverse events

	Concomitant tricuspid surgery (*n* = 910)	No tricuspid surgery (*n* = 167)	*P* value
In-hospital mortality	39 (4.3)	3 (1.8)	0.2
Stroke	24 (2.6)	3 (1.8)	0.8
Delirium	98 (12.1)	9 (6.3)	0.04
Intubation time (hours), median (IQR)	10 (5–20)	8 (5–14.5)	0.004
Re-exploration for bleeding	100 (11.3)	13 (8.8)	0.5
New-onset AF	68 (8.2)	23 (14.1)	0.03
Definitive PM	68 (8.1)	13 (8.6)	0.9
Low cardiac output	78 (8.6)	7 (4.3)	0.08
Postoperative dialysis	28 (3.5)	3 (2.1)	0.6
Vascular complications	14 (1.7)	8 (5.5)	<0.001
Thoracic wound complications	11 (1.2)	4 (2.5)	0.3
ICU stay (hours), median (IQR)	48 (21–120)	23 (20–47.4)	<0.001
Hospital stay (days), median (IQR)	11 (8–18)	8 (7–11)	<0.001

AF: Atrial fibrillation; IQR: interquartile range; PM: Pacemaker.

Stepwise backward multivariate logistic regression identified factors associated with the decision to perform concomitant TV repair (Table 4). The absence of severe TR was a key factor; patients with mild TR were significantly less likely to undergo TV repair (OR 4.06, 95% CI: 2.14–8.10, *P* < 0.001), as were those with moderate regurgitation (OR 3.31, 95% CI: 1.85–5.78, *P* < 0.001). Institutional practices also influenced decision-making, with centre-specific factors influencing the likelihood of TV repair (standard deviation for centre random intercept: 0.28, 95% CI: 0.18–0.39).

**Table 4:** Logistic regression analysis of preoperative factors influencing the decision against concomitant tricuspid valve repair

	*P* value	OR/SD	95% CI
Age	–	–	–
NYHA III-IV	0.02	0.61	0.34–0.92
Type of MV disease			
Degenerative (ref)	–	–	–
Functional	0.002	0.38	0.19–0.67
Rheumatic	0.004	0.34	0.16–0.74
Other	–	–	–
Tricuspid regurgitation			
Severe (ref)	–	–	–
Moderate	<0.001	3.31	1.85–5.78
Mild	<0.001	4.06	2.14–8.10
Tricuspid annular dilatation	<0.001	0.18	0.02–0.16
LVEF < 50%	–	–	–
Centres		0.28	0.18–0.39

Adjusted odds ratios with 95% CI are provided for each predictor. Centres were included as random intercept terms and SD and 95% were reported. CI: confidence interval; MV: mitral valve; OR: odds ratio.

**Figure 2:**
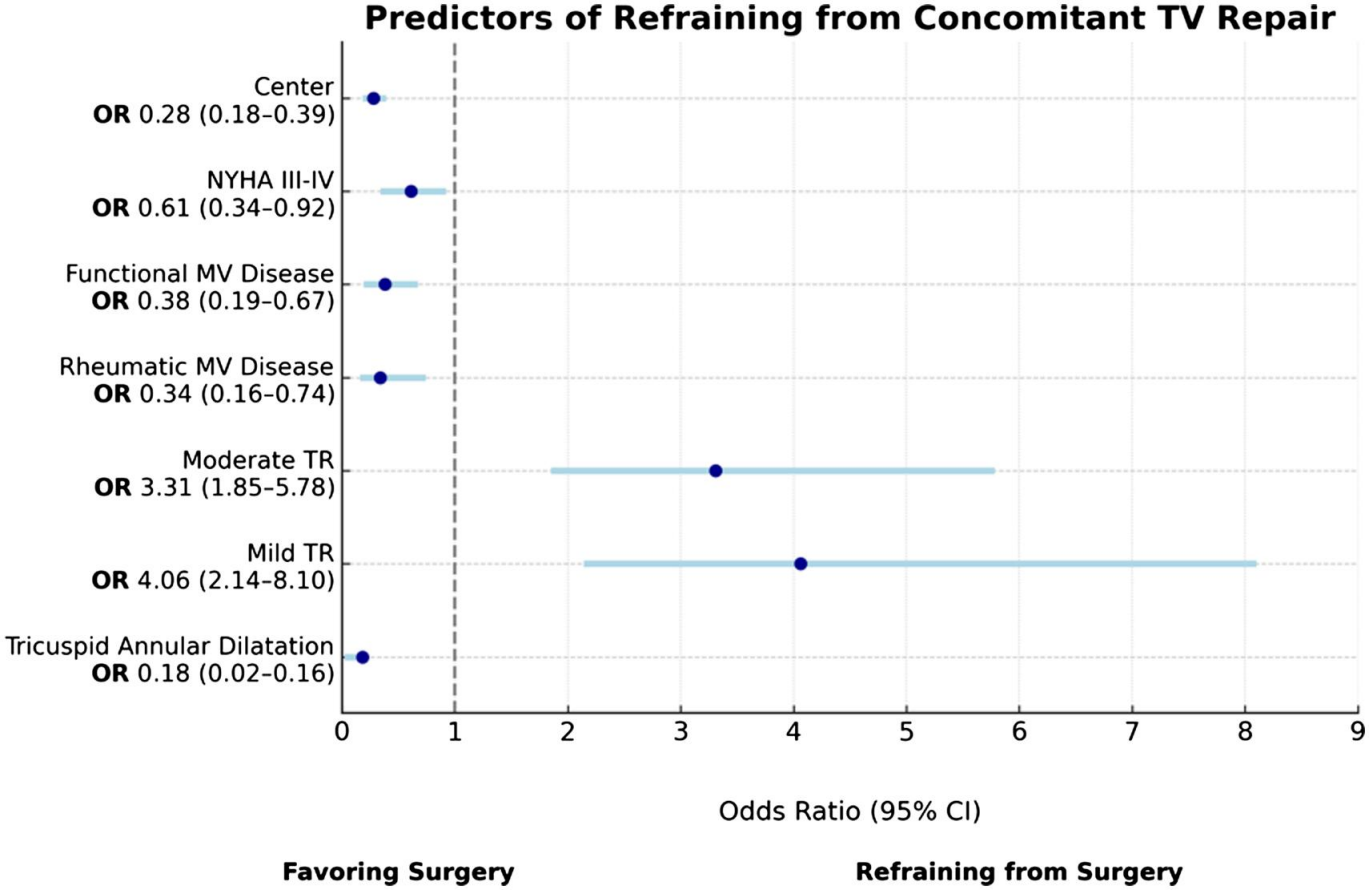
Forest plot. Forest plot depicting odds ratios (ORs) and 95% confidence intervals (CIs) for variables associated with the decision to omit tricuspid valve repair in patients undergoing minimally invasive mitral valve surgery. Key factors include tricuspid regurgitation severity, institutional practices and NYHA class.

Functional MV disease was associated with a lower likelihood of omitting TV repair compared to degenerative mitral disease (OR 0.38, 95% CI: 0.19–0.67, *P* = 0.002), as was rheumatic MV disease (OR 0.34, 95% CI: 0.16–0.74, *P* = 0.004), indicating that patients with functional mitral pathology were less frequently selected for tricuspid intervention. Conversely, significant tricuspid annular dilation strongly predicted TV repair (OR 0.18, 95% CI: 0.02–0.16, *P* < 0.001) (Fig. 2).

## DISCUSSION

This study highlights the nuanced decision-making involved in performing concomitant TV repair during MIMVS in patients meeting guideline-based criteria. We found that 84.5% of eligible patients underwent concomitant TV repair, primarily those with higher surgical risk profiles, such as advanced age, elevated EuroSCORE II and a higher prevalence of AFib. This trend suggests that surgeons prioritize TV repair in patients at higher risk of disease progression and right-sided heart failure, as addressing TV issues in these cases may reduce the likelihood of right ventricular dysfunction, improve haemodynamic stability and enhance survival and quality of life [10–12]. Interestingly, this observation contrasts with our initial hypothesis. We had expected that patients with greater illness severity might be less likely to receive concomitant TV repair due to concerns about increased procedural risk and operative burden. However, our data suggest the opposite—that surgeons may, in fact, favour a more comprehensive surgical strategy in higher-risk patients, possibly to prevent future clinical deterioration. Whether this is a conscious or rather intuitive choice remains a matter of debate.

However, this contrasts with our expected finding that high-risk patients might be less likely to undergo additional procedures. Our findings align with previous research indicating that concomitant TV repair during MIMVS is safe when performed in high-volume centres by experienced teams, offering patients the benefits of less invasive surgery without compromising outcomes [13]. Although patients who underwent TV repair had longer ICU and hospital stays, the absence of increased 30-day mortality suggests that the postoperative burden is mitigated by effective management and favourable short-term survival. These results indicate that with appropriate perioperative care, patients can receive comprehensive treatment without sacrificing safety.

Undoubtedly, patients selected for MIMVS undergo meticulous evaluation to ensure that both anatomical and clinical criteria for limited access surgery are met. The MMIR includes very experienced centres in minimally invasive atrioventricular valve surgery in which surgeons do not consider the presence of concomitant tricuspid disease as a contraindication for MIMVS. Surgery can be performed with the same high-quality and perioperative outcome as in an open setting [13]. Nevertheless, the presence of tricuspid disease has been related to worse long-term clinical outcomes probably reflecting a more advanced stage of the disease [14].

The decision not to perform TV repair in some patients, despite guideline recommendations, reveals a tension between theoretical benefits and practical limitations in real-world settings. In our study, institutional factors—including centre policies and individual surgeon preferences—significantly influenced the decision to perform TV repair. Variability in surgical practices reflects a tendency in some centres to avoid adding complexity to procedures, particularly where TV repair during MIMVS is uncommon, resources are limited, or TR severity is interpreted differently [15, 16]. Some surgeons may prioritize MV repair, especially in patients with primary mitral disease and lower NYHA functional class.

The relationship between MV and TV disease progression is complex and not fully understood [17]. Some surgeons might argue that TR may remain stable or improve after isolated MV repair, especially in patients without significant annular dilation or other risk factors [18]. However, the CTSNet trial provides evidence supporting concomitant tricuspid repair, further emphasizing the importance of current guidelines [7]. However, the major benefit demonstrated was related to patients with at least moderate TR at the presence of annular dilatation. On the other hand, patients with mild TR were less likely to benefit from the intervention. This fact is depicted in our study too, as it seems to be a taken into consideration for decision-making. According to our results, mild TR was less likely to be addressed than moderate or severe TR in MIMVS.

The type of underlying mitral pathology seems to play a role in decision-making regarding concomitant TV surgery. Functional mitral regurgitation is associated with either atrial, ventricular enlargement, or both, leading to mitral annular dilatation. This type of annular dilatation is commonly secondary to pre-existing conditions such as AFib or ischaemic and non-ischaemic cardiomyopathy. In the majority of the cases, all the conditions above affect both the mitral and the tricuspid annulus and lead to a similar degree of atrioventricular valve regurgitation. On the other hand, current echocardiography advancements have improved the diagnosis and initiation of treatment of primary MR. More patients are referred for MV repair nowadays at an early stage of the disease with no or limited signs of secondary effects such as TV pathology. Moreover, TV regurgitation improves in 30% of the patients undergoing successful repair for primary mitral regurgitation [19]. As the potential of TR improvement needs to be weighed on an individual patient basis, the risk of conduction abnormalities after concomitant repair may reduce its beneficial effect on TR progression.

Interestingly, aortic cross-clamp times were comparable between patients who underwent concomitant TV repair and those who did not. This observation may be explained by the variability in surgical technique across participating centres, with some tricuspid repairs performed on the beating heart and others under cardioplegic arrest. Furthermore, patients in the no-tricuspid repair group more commonly presented with degenerative MV disease, which often requires complex and time-intensive mitral repair procedures. These factors likely offset the expected increase in cross-clamp time associated with additional tricuspid intervention.

To provide context for our findings, we reviewed the literature reporting the rate of concomitant TV repair among patients undergoing open MV surgery. In a large-scale analysis of the Society of Thoracic Surgeons (STS) database comprising over 88 000 patients undergoing mitral operations between 2011 and 2014, the overall rate of concomitant TV repair was only 14%. Even among patients with a class I indication (severe TR), only ∼75.6% underwent TV repair. The corresponding figures were ∼30.6% for moderate TR and ∼3.5% for mild TR [20].

These findings are consistent with other reports demonstrating limited adherence to guideline recommendations in open surgery cohorts. For example, Kilic *et al.* [21] found that only ∼39% of patients with moderate TR received a concomitant repair, despite guideline-supported class IIa indications. These trends highlight a persistent underuse of tricuspid intervention, likely driven by concerns regarding added procedural complexity or risks such as pacemaker implantation.

In contrast, our study cohort—comprising patients undergoing MIMVS—demonstrated higher rates of concomitant tricuspid repair. Specifically, 89.1% of patients with severe TR (class I) and 81.4% of those with annular dilatation (class IIa) received a tricuspid repair. These rates are not only consistent with current guideline recommendations [22], but also surpass the rates reported in large open surgery series. These findings challenge the assumption that minimally invasive approaches lead to more conservative decision-making and instead suggest that, when performed in experienced centres, MIMVS can enable high compliance with guideline-directed care.

Interestingly, vascular complications were significantly more frequent in the group that did not undergo concomitant TV repair. This finding may reflect institutional or technical factors related to the surgical approach, particularly in the context of MIMVS, where peripheral cannulation is commonly employed. It is known that percutaneous femoral cannulation, while facilitating rapid access and avoiding surgical cut-down, may be associated with a higher incidence of vascular complications, particularly in less experienced centres or in patients with challenging anatomy. In contrast, surgical femoral cut-down may offer more controlled vessel exposure and potentially reduce access-related complications. The observed difference in vascular event rates could therefore be influenced by both cannulation strategy and the learning curve or procedural volume of the participating centres. These results highlight the importance of cannulation technique, careful patient selection and centre experience in optimizing outcomes in MIMVS.

Notably, we observed no significant difference in the rate of permanent pacemaker implantation between patients who underwent concomitant TV repair and those who did not. This is an important finding, as concerns about conduction disturbances and subsequent need for pacemaker implantation have historically contributed to the hesitancy in performing tricuspid interventions. Our results suggest that, within the context of experienced centres and contemporary surgical techniques, the addition of tricuspid repair does not increase the risk of conduction-related complications requiring permanent pacing. This supports the procedural safety of concomitant tricuspid intervention in appropriately selected patients.

Ultimately, the decision to perform concomitant TV repair is up to the surgeon. In this study, we hypothesized that decision-making was based on an institutional policy, especially when considering patients with softer indications. Despite the expertise and high MIMVS volumes in the MMIR registry centres, a small percentage of patients with clear indications did not receive TV surgery—often those with higher illness severity. The TRIGISTRY report shows that patients with early-stage TV disease may benefit from early surgical intervention to prevent right ventricular damage and secondary organ failure [23]. Extending this approach to lower-risk patients with indicated TV disease could be beneficial, as concomitant TV surgery does not significantly increase operative time or perioperative mortality.

### Limitations

As a retrospective observational study, ours is subject to selection bias, as the decision to perform TV repair may have been influenced by unmeasured patient or institutional factors. These patients were highly selected, with the most severely ill excluded from MIMVS. The observational nature of the study limits our ability to infer causation, as associations observed may not indicate direct effects. The multicentre design also introduces variability in protocols, expertise and resources, which may affect outcomes and limit generalizability. We focused primarily on short-term outcomes, such as ICU and hospital stays and 30-day mortality, without examining long-term outcomes, such as quality of life, TR progression and right ventricular function. Additionally, we did not account for the influence of surgeon experience or centre volume, both of which could impact outcomes in MIMVS and concomitant TV repair. Future research should address these limitations with prospective studies, long-term follow-up and standardized protocols to provide a clearer picture of the impact of concomitant TV repair. Another limitation of our study is the use of a traditional 4-grade scale for TR severity. Although this classification was widely accepted during much of the study period, a 6-grade scale has more recently gained traction for its enhanced granularity and clinical relevance. The use of the older grading system may have limited the precision of TR severity assessment and should be considered when interpreting the results.

## CONCLUSION

Our findings highlight that the decision to perform concomitant TV repair in MIMVS is influenced by a combination of patient-related and institutional factors. While in-hospital outcomes were similar between patients who did and did not receive TV repair, these findings primarily reflect technical and short-term procedural success. As our study does not include long-term follow-up, the impact of performing or omitting TV repair on clinical outcomes over time remains unclear. Future studies with extended follow-up are warranted to determine whether adherence to guideline-based indications for TV surgery translates into improved long-term outcomes.

## FUNDING

None declared.

**Conflict of interest:** Nikolaos Bonaros had speakers honoraria by Edwards Lifesciences, Medtronic, institutional grant by Edwards Lifesciences, Corcym; and travel Grants by Abbott, Medtronic, Edwards Lifesciences. Jörg Kempfert had speaker honoraria by Edwards Lifesciences, Medtronic, Artivion, Abbott. Antonios Pitsis had speaker honoraria by Medtronic, Edwards Lifesciences, Delacroix Chevalier. Can Gollmann-Tepeköylü had speakers honoraria by Edwards Lifesciences. The other authors have no conflicts of interest to declare.

## Data Availability

The data underlying this article will be shared on reasonable request to the corresponding author.

## ABBREVIATIONS

AFib: Atrial fibrillation
CI: Confidence interval
CPB: Cardiopulmonary bypass
MIMVS: Minimally invasive mitral valve surgery
MMIR: Mini Mitral International Registry
MV: Mitral valve
OR: Odds ratio
TR: Tricuspid regurgitation
TV: Tricuspid valve

## References

[1] Santana O , Larrauri-Reyes M, Zamora C, Mihos CG. Is a minimally invasive approach for mitral valve surgery more cost-effective than median sternotomy? Interact CardioVasc Thorac Surg 2016;22:97–100. 26433974 10.1093/icvts/ivv269

[2] Seeburger J , Borger MA, Falk V et al Minimal invasive mitral valve repair for mitral regurgitation: results of 1339 consecutive patients. Eur J Cardiothorac Surg 2008;34:760–5. 18586512 10.1016/j.ejcts.2008.05.015

[3] Ko K , de Kroon TL, Post MC et al Minimally invasive mitral valve surgery: a systematic safety analysis. Open Heart 2020;7. 10.1136/openhrt-2020-001393 PMC7552840 33046594

[4] Bolumburu AA , Ruiz JMM, Mahia P et al Determinants of tricuspid regurgitation progression and its implications for adequate management. JACC Cardiovasc Imaging 2024;17:579–91. 38069980 10.1016/j.jcmg.2023.10.006

[5] Nishimura RA , Otto CM, Bonow RO et al 2017 AHA/ACC focused update of the 2014 AHA/ACC guideline for the management of patients with valvular heart disease: a report of the American College of Cardiology/American Heart Association Task Force on Clinical Practice Guidelines. Circulation 2017;135:e1159–95. 28298458 10.1161/CIR.0000000000000503

[6] Vahanian A , Beyersdorf F, Praz F et al; ESC/EACTS Scientific Document Group. 2021 ESC/EACTS guidelines for the management of valvular heart disease. Eur Heart J 2022;43:561–632. 34453165 10.1093/eurheartj/ehab395

[7] Gammie JS , Chu MWA, Falk V et al; CTSN Investigators. Concomitant tricuspid repair in patients with degenerative mitral regurgitation. N Engl J Med 2022;386:327–39. 34767705 10.1056/NEJMoa2115961 PMC8796794

[8] Tornos Mas P , Rodríguez-Palomares JF, Antunes MJ. Secondary tricuspid valve regurgitation: a forgotten entity. Heart 2015;101:1840–8. 26503944 10.1136/heartjnl-2014-307252 PMC4680164

[9] Berretta P , Kempfert J, Van Praet F et al Risk-related clinical outcomes after minimally invasive mitral valve surgery: insights from the Mini-Mitral International Registry. Eur J Cardiothorac Surg 2023;63:ezad090. 10.1093/ejcts/ezad090 36892446

[10] Thourani VH , Bonnell L, Wyler von Ballmoos MC et al Outcomes of isolated tricuspid valve surgery: a Society of Thoracic Surgeons analysis and risk model. Ann Thorac Surg 2024;118:873–81. 38723881 10.1016/j.athoracsur.2024.04.014

[11] McCarthy PM , Bhudia SK, Rajeswaran J et al Tricuspid valve repair: durability and risk factors for failure. J Thorac Cardiovasc Surg 2004;127:674–85. 15001895 10.1016/j.jtcvs.2003.11.019

[12] Pfannmüller B , Davierwala P, Hirnle G et al Concomitant tricuspid valve repair in patients with minimally invasive mitral valve surgery. Ann Cardiothorac Surg 2013;2:758–64. 24349978 10.3978/j.issn.2225-319X.2013.10.01 PMC3856997

[13] Badhwar V , Rankin JS, He M et al Performing concomitant tricuspid valve repair at the time of mitral valve operations is not associated with increased operative mortality. Ann Thorac Surg 2017;103:587–93. 27570159 10.1016/j.athoracsur.2016.06.004

[14] Bonaros N , Hoefer D, Oezpeker C et al Predictors of safety and success in minimally invasive surgery for degenerative mitral disease. Eur J Cardiothorac Surg 2022;61:637–44. 34738105 10.1093/ejcts/ezab438

[15] Arroyo NA , Gessert T, Hitchcock M et al What promotes surgeon practice change? A scoping review of innovation adoption in surgical practice. Ann Surg 2021;273:474–82. 33055590 10.1097/SLA.0000000000004355 PMC10777662

[16] Adams DH , Rosenhek R, Falk V. Degenerative mitral valve regurgitation: best practice revolution. Eur Heart J 2010;31:1958–66. 20624767 10.1093/eurheartj/ehq222 PMC2921508

[17] Shiran A , Sagie A. Tricuspid regurgitation in mitral valve disease: incidence, prognostic implications, mechanism, and management. J Am Coll Cardiol 2009;53:401–8. 19179197 10.1016/j.jacc.2008.09.048

[18] Katsi V , Raftopoulos L, Aggeli C et al Tricuspid regurgitation after successful mitral valve surgery. Interact CardioVasc Thorac Surg 2012;15:102–8. 22457188 10.1093/icvts/ivs107 PMC3380985

[19] Hage A , Hage F, Jones PM, Manian U, Tzemos N, Chu MWA. Evolution of tricuspid regurgitation after repair of degenerative mitral regurgitation. Ann Thorac Surg 2020;109:1350–5. 31545970 10.1016/j.athoracsur.2019.08.025

[20] Chikwe J , Itagaki S, Anyanwu A, Adams DH. Impact of concomitant tricuspid annuloplasty on tricuspid regurgitation, right ventricular function, and pulmonary artery hypertension after repair of mitral valve prolapse. J Am Coll Cardiol 2015;65:1931–8. 25936265 10.1016/j.jacc.2015.01.059

[21] Kilic A , Saha-Chaudhuri P, Rankin JS, Conte JV. Trends and outcomes of tricuspid valve surgery in North America: an analysis of more than 50,000 patients from the Society of Thoracic Surgeons database. Ann Thorac Surg 2013;96:1546–52;discussion 1552. 24070702 10.1016/j.athoracsur.2013.06.031

[22] Nishimura RA , Otto CM, Bonow RO, Carabello BA, Erwin JP 3rd, Guyton RA et al 2014 AHA/ACC guideline for the management of patients with valvular heart disease: a report of the American College of Cardiology/American Heart Association Task Force on practice guidelines. J Am Coll Cardiol 2014;63:e57–185. 24603191 10.1016/j.jacc.2014.02.536

[23] Dreyfus J , Galloo X, Taramasso M et al; TRIGISTRY Investigators. TRI-SCORE and benefit of intervention in patients with severe tricuspid regurgitation. Eur Heart J 2024;45:586–97. 37624856 10.1093/eurheartj/ehad585

